# An Orthogonal Supramolecular Approach toward Protein Binding and Protein Sensing Using Dendrimers as Scaffolds for the Noncovalent Assembly of Binding and Sensing Groups

**DOI:** 10.1021/acsmaterialsau.5c00049

**Published:** 2025-08-08

**Authors:** Azrah Aziz, Lance J. Twyman, Amal Al Ageel, Ibrahim O. Althobaiti, Abdullah N. Alotaibi

**Affiliations:** School of Mathematical and Physical Sciences, Chemistry, University of Sheffield, Dainton Building, Brook Hill, Sheffield, South Yorkshire S3 7HF, U.K.

**Keywords:** protein binding, protein−protein binding, dendrimers, self-assembly, supramolecular materials, dynamic materials

## Abstract

Inhibiting unwanted protein–protein interactions (PPIs) by targeting extensive protein binding surfaces presents a significant challenge. Macro-ligands offer a promising approach, but traditional covalent functionalization strategies often suffer from synthetic complexity, particularly in controlling the spatial arrangement of binding moieties. This study introduces a new method for macro-ligand design based on the noncovalent, modular self-assembly of functional units within an inert dendrimer scaffold. Although these units are embedded within the dendrimer in a random arrangement, they are mobile and free to move. As such, when a target protein is introduced, these binding units can undergo a self-organization process to optimize their spatial distribution and maximize cooperative interactions with the protein’s binding surface. This dynamic process is controlled by the protein, as it guides and controls the formation of its own optimized macromolecular ligand. When sensor units are combined and included in the assembly process, real-time monitoring and quantification of binding can be detected and quantified. This study details the synthetic methodology employed for the preparation of the component parts and their self-assembly into dendrimer complexes. Subsequent binding assays using cytochrome-c as the target protein, and associated dendrimer complexes, exhibited binding affinities in the nanomolar (nM) range.

## Graphical abstract

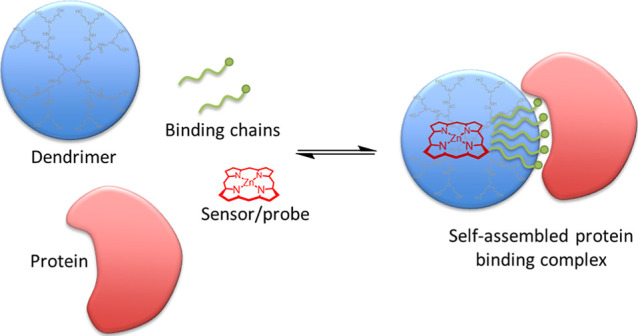

## Introduction

The complexes formed when proteins interact with other proteins^1^ or biological macromolecules^2,3^ play essential roles in all biological processes. Unwanted or uncontrolled interactions, as well as conditions leading to protein mutations, often result in disease.^4^ These include neurodegenerative diseases such as Alzheimer’s and Huntington’s disease.^5^ Protein–protein interactions are also involved in viral and bacterial infections. For example, binding between proteins on the surface of bacteria and cells can facilitate internalization (of the bacteria) within the host cell.^6^ Using a similar mechanism, it has been demonstrated that viral proteins can bind to host proteins, resulting in internalization and infection.^7^ As such, understanding how to modulate or inhibit protein–protein interactions is an emerging concept in drug design.

Proteins recognize each other through complementary functionalities positioned at precise points on large interacting surfaces that can range from 500 Å^2^ to 5000 Å^2^; the key component of which is known as the “hot spot” or interfacial area.^8^ Therefore, one challenge in designing inhibitors is the construction of architectures that are large enough to interact with most, or all, of the interfacial area of a protein.^9^ As well as size, an array of noncovalent interactions are also important with respect to an inhibitor’s selectivity, including charge/charge, hydrophobic, aromatic/π–π interactions and hydrogen bonding.^10^ Studies into protein–protein interactions have identified specific amino acids that consistently contribute more than 2 kcal/mol to the binding energy, while appearing at the interfacial surface with a frequency greater than 10%.^11^ These amino acids are capable of making multiple interactions and include; tryptophan 21%, arginine 14% and tyrosine 13%. As such, multi/polyvalency, functionality, charge and size are key design determinants with respect to obtaining selective ligands for protein binding. Given these requirements it is not surprising that macromolecular ligands show great promise with regards to protein binding. Examples include calixarene and porphyrin scaffolds,^12,13^ nanomaterials,^14^ and linear polymers.^15,16^ We have studied the use of dendrimers and functionalized dendrimers as protein binding ligands.^17,18^ Our preliminary studies involved a series of negatively charged carboxylate dendrimers designed to interact with proteins possessing positively charged interfacial areas of various sizes. The results revealed that selective binding could be predicted and achieved by matching the dendrimer’s size/maximum addressable area, with the size of a protein’s interfacial or binding area.^17^ We then investigated the importance of terminal group functionality on binding. Binding experiments indicated that a tyrosine functionalized dendrimer bound chymotrypsin with a relative affinity 30% stronger than an unfunctionalized dendrimer of similar size and charge.^19^ These results were further extended and quantified, using porphyrin cored dendrimers and fluorescence titrations.^20^ Overall, these experiments demonstrated the importance of size and functionality when designing macromolecular ligands for selective protein binding. Although these results were successful and demonstrated a clear proof of principle, covalent chemistry is time-consuming with respect to incorporation of specific terminal groups and core functionality. In addition, errors in design or synthesis cannot be corrected easily, requiring a new macromolecular/dendrimer ligand to be synthesized. Furthermore, although adding a *single*/*specific* functional group many times to the surface of the dendrimer is relatively easy (polyvalency), it is extremely difficult to position such moieties in respect to each other with geometric precision. It is even more difficult to add a number of *different* functional groups (multivalency) with control regarding their relative 3D position to each other: Yet any therapeutically useful macromolecule will require such precision in its design.

This paper describes our initial “proof of concept” methodology that attempts to address these aims. The approach uses a noncovalent methodology for the assembly of targeting and sensing units within and around a dendrimer scaffold. Control over the relative position of targeting groups can be achieved using a target protein as a template, directing formation of an optimized macromolecular protein complex through various reversible cooperative interactions. The design and methodology are shown schematically in Figure 1. The dendrimer, its binding units and a sensing component are added to water, where they can assemble into the random complex **1**. When a protein is added, the randomly distributed binding units are free to move within the dendrimer so that they maximize any cooperative protein-binding interactions. Binding can then be detected and quantified via perturbation of an encapsulated sensing group’s photophysical properties.

**Figure 1:**
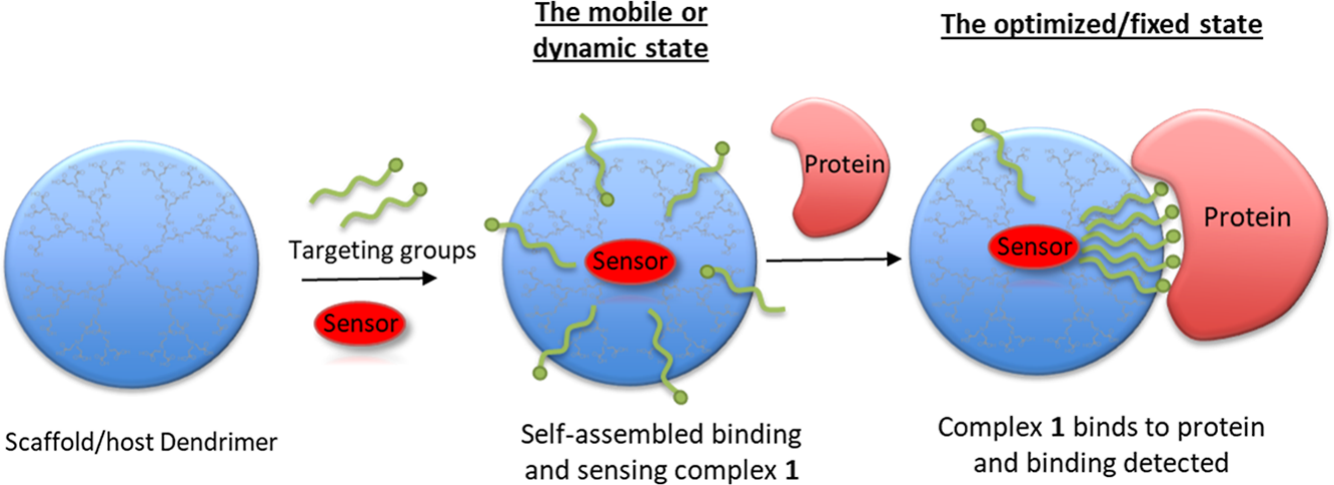
Schematic showing the proposed self-assembled protein binding complex **1** and its binding to a target protein. A dendrimer acts as a scaffold to support and encapsulate the binding and sensing units. The use of noncovalent chemistry allows the targeting groups to move and maximize their binding efficiency in the presence of a target protein.

This paper describes a dynamic noncovalent approach for measuring and enhancing dendrimer–protein binding by simultaneously functionalizing the dendrimer with sensor and protein-binding units. We further explore how this methodology can be adapted for dendrimers that lack intrinsic protein-binding capabilities. In both instances, the dendrimer serves as a scaffold, supporting various components that facilitate binding to a target protein, while also offering a mechanism to detect and quantify this binding.

## Results and Discussion

### Selection of the Protein and Dendrimer Components

The proposed approach uses noncovalent chemistry to assemble an optimized macromolecular ligand using a target protein as a template. This design concept requires three separate components as shown in Figure 2. With respect to the dendrimer, it must be water-soluble, to ensure that the protein and the assembled ligand are in the same aqueous phase. Additionally, the dendrimer needs to be large enough to address the target protein’s binding/interfacial area, while also ensuring that incorporation of the binding/sensing units is not prevented or limited by a dense shell or dense packed structure.^21,22^ For this work, we selected cytochrome-c as the target protein. The structure of this protein is well-known, having a binding interface (hot-spot) that is rich in positive charge and is around 1100 Å^2^ in size.^23^ We have previously demonstrated that a G2.5 and G 3.5 dendrimer, with 16 and 32 terminal carboxylic acid groups respectively, could bind effectively to this protein using electrostatic interactions.^24^ In addition, cytochrome *c* possess a porphyrin unit, which will enable binding to be studied using fluorescence and emission spectroscopy. For the work described herein, we selected and synthesized the larger G3.5 CO_2_H dendrimer **2**, as its size and packing density are optimal for maximum guest encapsulation.^17,25,26^ Although the G3.5 CO_2_H dendrimer **2** can interact with cytochrome *c* through a number of cooperative simple charge–charge interactions,^27^ the inclusion of additional units that present specific binding groups on the dendrimer’s surface will further enhance binding. However, to fully demonstrate the applicability of the proposed methodology, we also proposed the use of the hydroxyl terminated G 4.0-OH **3** dendrimers.^24,26^ Although a higher generation, this dendrimer has a similar size to the G3.5 CO_2_H dendrimer **2**. In addition, at neutral pH the OH groups on the outside of G 4.0-OH **3** do not dissociate, and the dendrimer is neutral. As such, the G 4.0-OH **3** dendrimers are unlikely to bind to cytochrome *c* in the absence of additional functionality. Both target dendrimers were synthesized using well-known and published procedures.^17,26^

**Figure 2:**
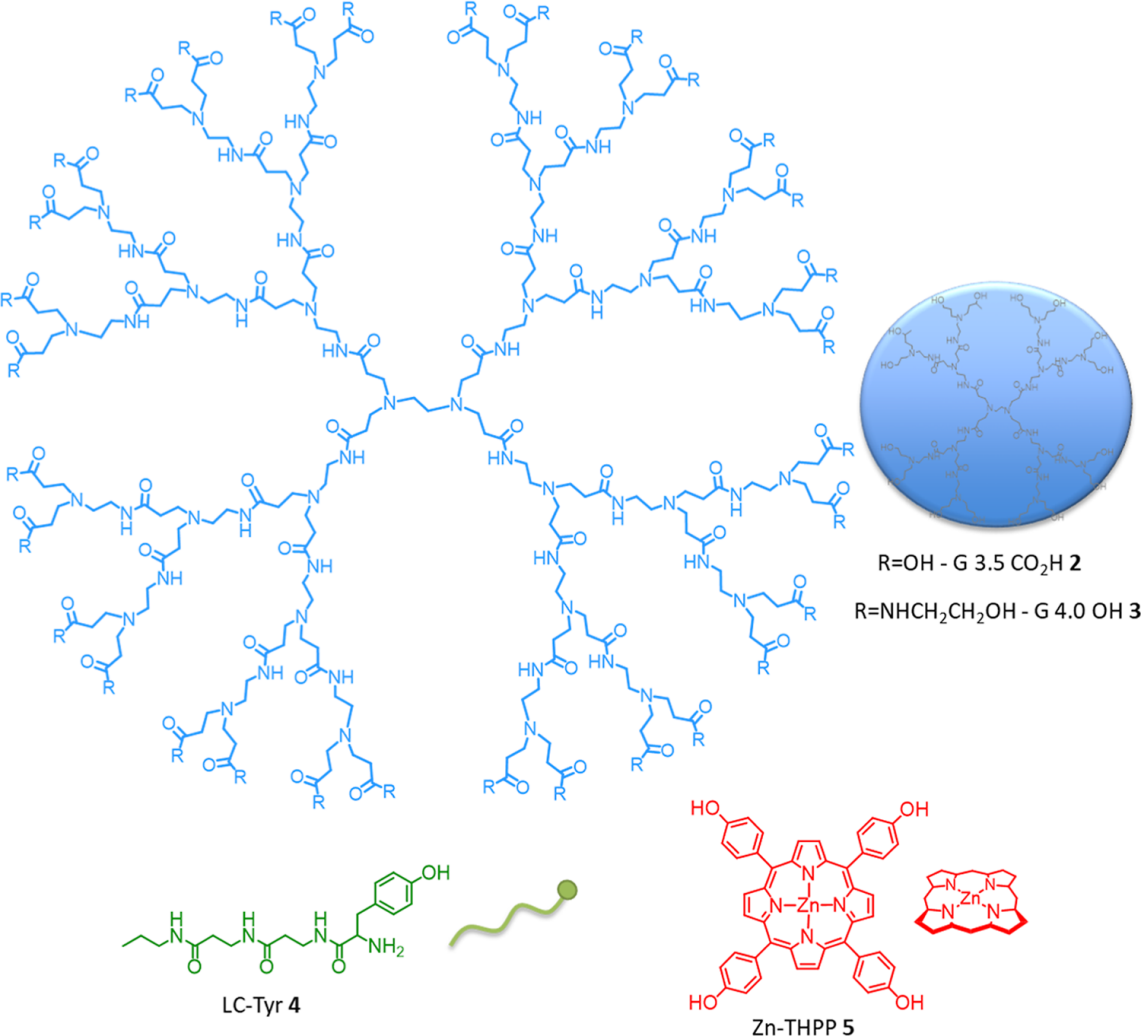
The structure and schematic representations of the three components required for the self-assembled macromolecular protein ligand **1**.

### Synthesis of the Linear Chain Component

The second component was a targeting/binding unit designed for encapsulation within the dendrimer’s interior, while presenting a binding group on the dendrimer’s surface. This was achieved by functionalizing a hydrophobic oligomeric amide chain with an amino acid targeting group.^13^ The chain’s design incorporated cooperative hydrophobic and hydrogen-bonding interactions to promote encapsulation. Tyrosine–tyrosine interactions are known to be important for protein–protein binding, contributing to both affinity and specificity.^28^ Furthermore, the surface of cytochrome *c* and its complexes has been mapped and there are a number of key tyrosine units’ positioned on the binding surfaces.^29^ As such, the tyrosine linear chain **4** (LC-Tyr **4**) was chosen and synthesized as shown in Scheme 1. The synthesis began with propyl amine **6**, followed by stepwise addition of two β-alanine repeat units **7** and subsequent deprotection, yielding amine-terminated chain **11**. Finally, benzyloxy-protected tyrosine was added to give the protected chain **12** which was subsequently deprotected to give the target linear chain, LC-Tyr **4**, in good yield.

**Scheme 1:**
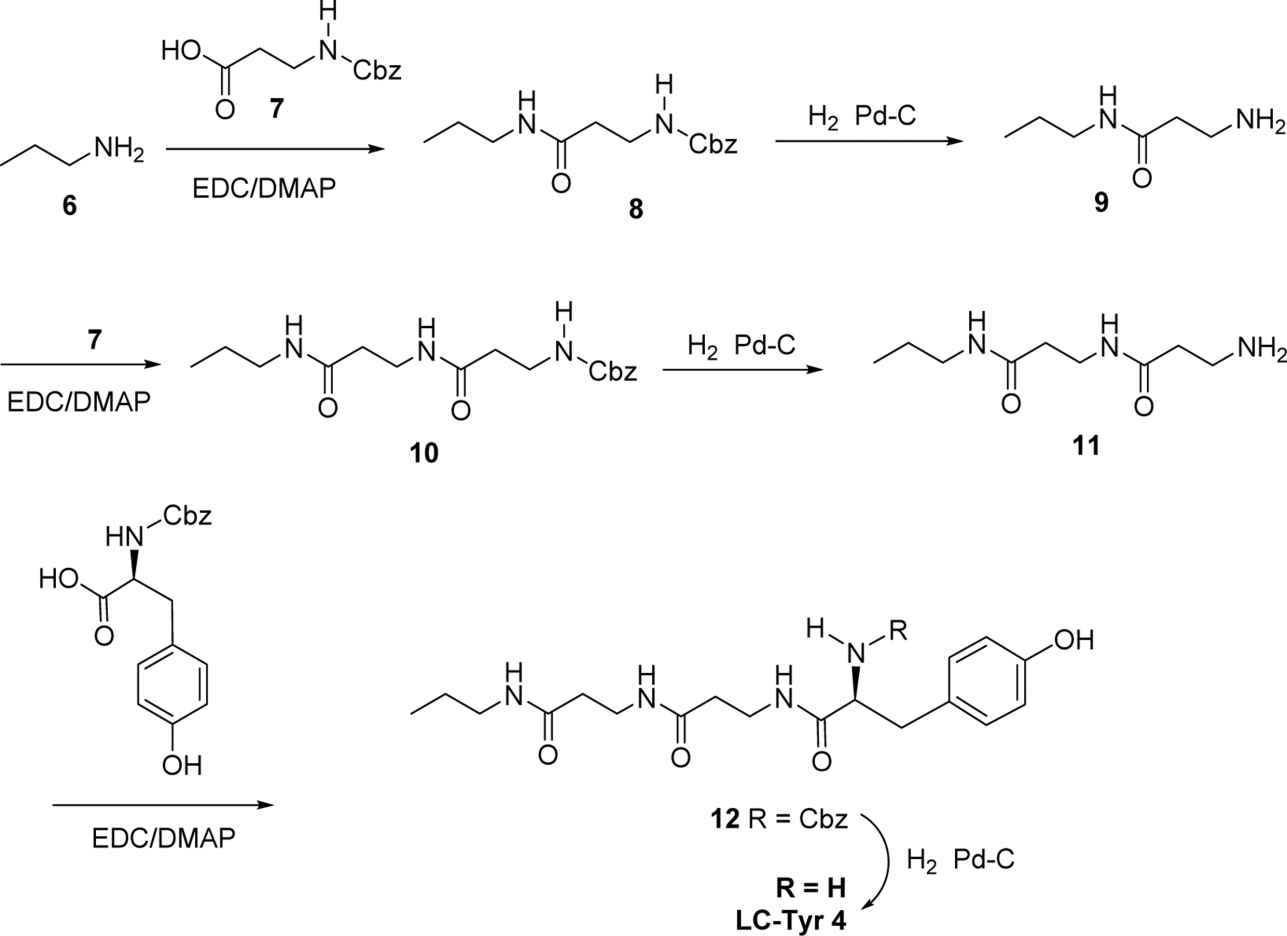
Synthesis of the Tyrosine Terminated Linear Chain LC-Ty **4**

### Synthesis of the Porphyrin Sensor Component

The final component of the self-assembled protein ligand system **1** is a simple porphyrin that can be used as the sensing/detection unit. As well as possessing a well-characterized binding area and a size compatible with the scaffold G 4.0 OH **2** and G 3.5 CO_2_H **3** dendrimers, cytochrome *c* is a porphyrin-containing protein that emits a strong fluorescence signal that can be perturbed by a bound quencher.^12,17,19^ This property can be exploited to detect and quantify binding using a hydrophobic quencher encapsulated within the interior of the dendrimer. It is essential that this quencher does not bind to the protein independently and is retained within the dendrimer. As such, zinc-tetra­(4-hydroxyphenyl) porphyrin **5** (Zn-THPP) was selected as the internal quencher. Zn-THPP **5** is almost insoluble in water,^30^ and can be encapsulated within the dendrimer using simple hydrophobic interactions.^26^ In addition, the zinc atom at the center of porphyrin **5** can coordinate to the dendrimer’s internal amines, which strengthens binding and encapsulation. Porphyrin **5** also has four phenolic OH groups that can hydrogen bond to the dendrimer’s amides. In addition, the OH groups are acidic enough to be deprotonated by the internal amines, resulting in additional electrostatic interactions.^26^ As such, there are a number of cooperative interactions that will help ensure porphyrin **5** stays encapsulated within the scaffold dendrimers **2** and **3**. Zn-THPP **5** was obtained easily in two steps, starting from 4-hydroxybenzaldehyde and pyrrole, to give the initial tetra­(4-hydroxyphenyl)­porphyrin **13**, before insertion of zinc to give the final Zn-THPP **5** in a yield of 3% after recrystallizations, Scheme 2.

**Scheme 2:**
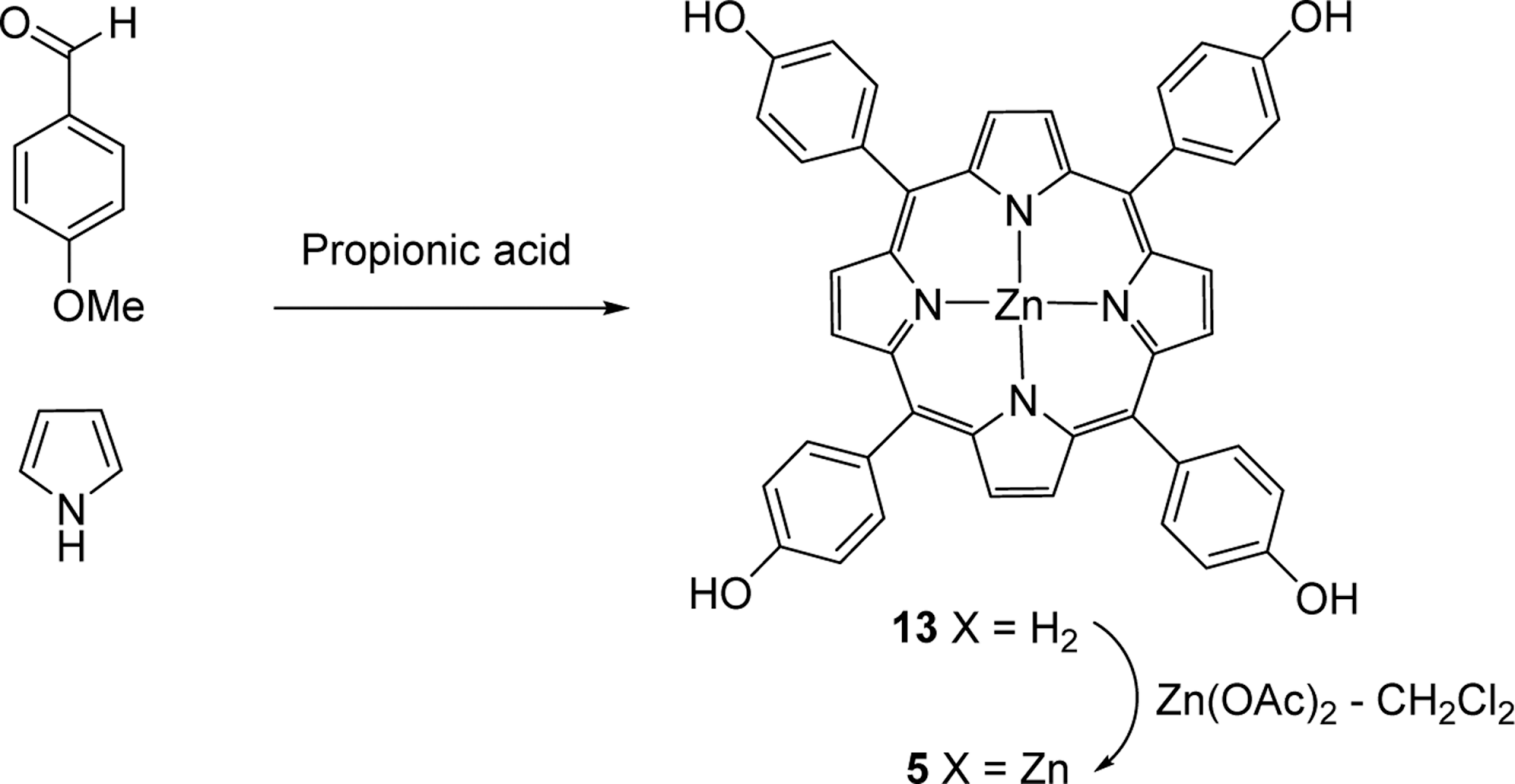
Synthesis of the Zinc Tetrahydroxyphenyl Porphyrin **5** (ZnTHPP) Sensor

### Encapsulation of the Linear Chain and Sensor Units

Encapsulation experiments to determine the loading potential of LC-Tyr **4** within the G 3.5-CO_2_H **2** and G 4.0 OH **3** dendrimers were achieved using a procedure similar to that used to solubilize and encapsulate hydrophobic drugs within water-soluble PAMAM dendrimers.^26^ Specifically, 11 equiv of LC-Tyr **4** were added to methanol solutions of the G 3.5-CO_2_H **2** and G 4.0 OH **3** dendrimers. In each case the methanol was removed and a known volume of buffer (0.01 M PBS, pH 7.4) added to give a final dendrimer concentration of 1 × 10^–6^ M. The solutions were then filtered to remove any undissolved material and analyzed by UV to determine the concentration of LC-Tyr **4** (for both dendrimers). Beer–Lambert analysis confirmed that all 11 linear chains had been dissolved/encapsulated, giving a final concentration of 1.1 × 10^–5^ M for LC-Tyr **4** in solutions of both dendrimers **2** and **3**. This is higher than the solubility of LC-Tyr **4** without the dendrimer, which had a maximum solubility 0.51 × 10^–5^ M in buffer. The difference between these concentrations indicates that at least 6 LC-Tyr **4** are encapsulated within the dendrimers **2** and **3**. As encapsulation is driven by cooperative hydrophobic and H-bonding interactions (in addition to salt formation for the carboxylic acid dendrimers), it is probable that all the linear chains were encapsulated within the dendrimer. This was supported by a 5 nm bathochromic shift in λ_max_ when LC-Tyr **4** was encapsulated within dendrimers **2** and **3** and is consistent with a change in environment (peak shifted from 270 to 275 nm).^31^ While the precise orientation of the bound LC-Tyr **4** within the dendrimer is not known, it seemed logical to assume that the hydrophobic tail would favor a position closer to the dendrimer’s hydrophobic core, Figure 3. Although LC-Tyr **4** could potentially bind with the tyrosine head buried within the dendrimer, the dynamic nature of the proposed approach would allow LC-Tyr **4** to reorient itself for optimal binding to the target protein as shown in Figure 1. A similar procedure was used to encapsulate one equivalent of the porphyrin-signaling unit Zn-THPP **5**, which exhibited extremely low aqueous solubility and could barely be detected by UV. After encapsulation of Zn-THPP **5**, there was no evidence of any porphyrin precipitated and encapsulation and binding were confirmed by UV analysis, which indicated a strong Soret band that had shifted from 412 to 425 nm. This shift is a characteristic of complexation (between zinc and nitrogen) and confirms encapsulation within the dendrimer’s interior.^32^ Having established and quantified encapsulation of the various components, the next step involved the key series of binding experiments to test whether the self-assembled complex could bind to cytochrome *c* and be detected.

**Figure 3:**
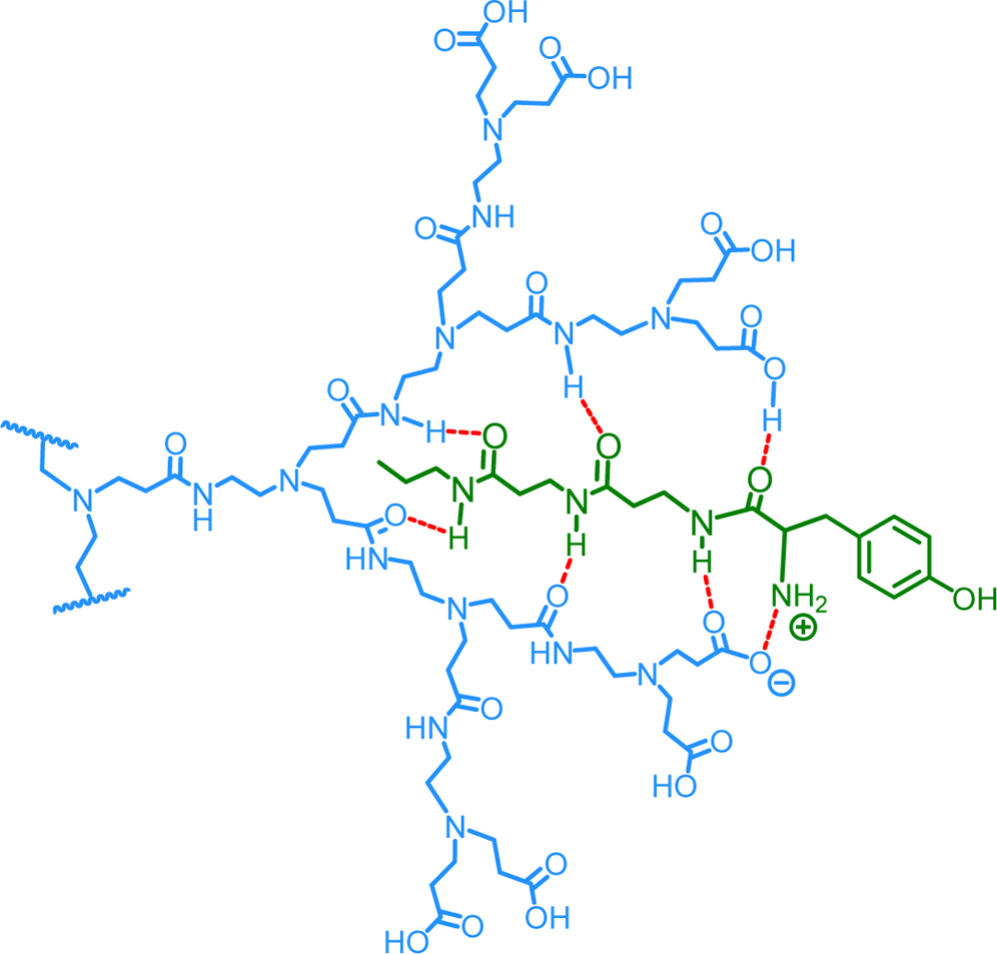
Schematic showing a segment of the G3.5-CO_2_H dendrimer **2** and possible H-bonding and salt formation to the linear chain, LC-Tyr **4**.

### Protein Binding Using a Carboxylic Acid Terminated Dendrimer and Amino Acid Complex

Our first experiment was a control using the carboxylic acid terminated dendrimer G3.5-CO_2_H **2** and the sensing unit Zn-THPP **5**. The LC-Tyr **4** was not included in this experiment. This would allow us to obtain a baseline for comparison with data obtained in later experiments. The experiment was carried out at pH 7.4 by titrating a solution of cytochrome *c* into a solution of the dendrimer complex, assembled from a 1:1 ratio for G3.5-CO_2_H dendrimer **2** and Zn-THPP **5** (1 × 10^–6^ M for both species **2** and **5**). Detection and quantification of binding was achieved by following changes to the intensity of the Zn-THPP **5** emission band at 610 nm, as it is quenched by bound cytochrome *c*.^17^ The change in intensity was plotted with respect to cytochrome *c* concentration, and a dissociation constant (*K*_d_) of 11 nM was obtained by fitting the experimental data to a 1:1 binding model, Figure 4. The experiment was then repeated with the inclusion of the functionalized chain, LC-Tyr **4**. In this experiment, a 1:1:11 ratio of G3.5-CO_2_H **2** dendrimer, Zn-THPP **5** and LC-Tyr **4** was used (1 × 10^–6^ M for both G3.5-CO_2_H **2** dendrimer and Zn-THPP **5** and 1.1 × 10^–5^ M for LC-Tyr **4**). As before, the change in intensity vs cytochrome-c concentration was plotted and the experimental data fitted to a 1:1 binding model, Figure 4. On this occasion, a *K*_d_ of 5.5 nM was obtained, indicating a 100% increase in binding affinity for the functionalized system. These results clearly supported our hypothesis that a simple self-assembly method could be used to generate functionalized dendrimers, and that these dendrimers could interact with a protein with a higher affinity than the corresponding nonfunctionalized dendrimer. Furthermore, the noncovalent encapsulation of a sensing moiety enabled quantification of this interaction.

**Figure 4:**
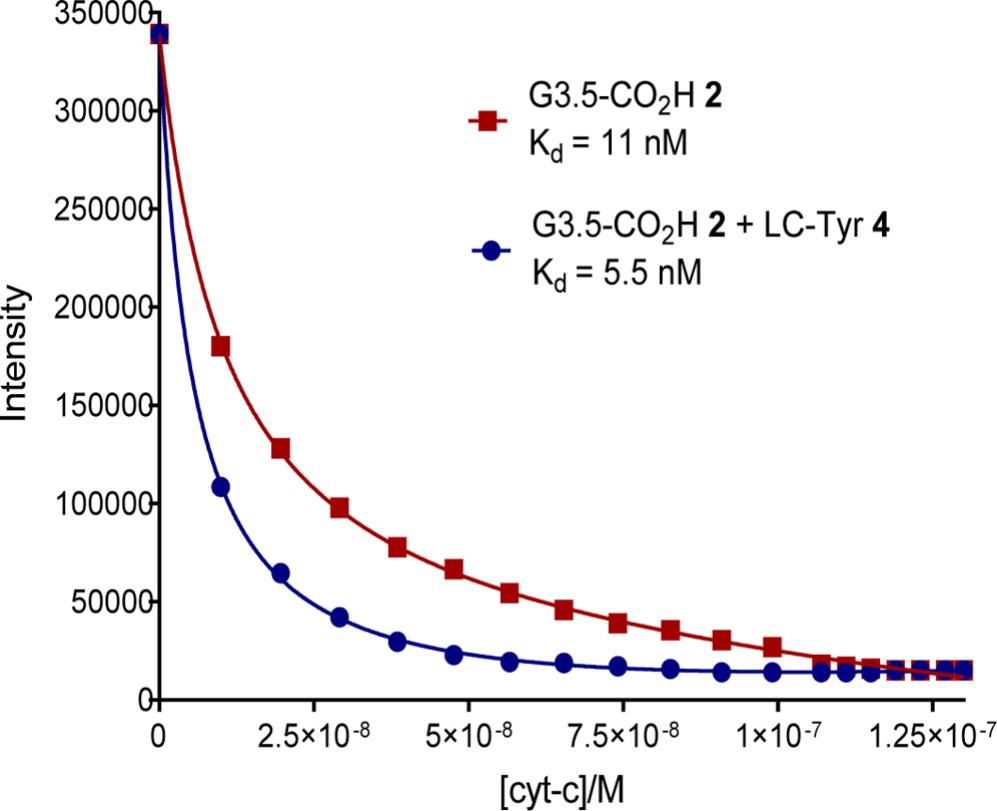
Titration plots of cytochrome *c* and the unfunctionalized complex using dendrimer **2** (square points). In this case binding is due solely to electrostatic interactions between the dendrimer’s terminal groups and the protein’s binding area. When the tyrosine linear chain (LC_Tyr **4**) is added to give complex **1**, the binding is significantly strengthened by additional interactions between tyrosine’s aromatic, phenolic and amide functional groups (circle points). Plots show changes of intensity for the encapsulated Zn-THPP **5** peak at 615 nm (excitation at 410 nm) as cytochrome *c* is added.

### Protein Binding Using Neutral Non-binding Dendrimers and Amino Acid Complex

To further investigate and challenge the proposed methodology, an additional experiment was conducted using a neutral dendrimer G4.0-OH **3**. This dendrimer does not possess any terminal charges and is incapable of binding the target protein in the absence of any additional functionality. The experiment followed the same protocol described above, employing a 1:1:11 molar ratio of G 4.0-OH **3** dendrimer, Zn-THPP **5**, and LC-Tyr **4** (1 × 10^–6^ M for both G 4.0-OH **3** and Zn-THPP **5**, and 1.1 × 10^–5^ M for LC-Tyr **4**).

Upon titration and increasing concentrations of cytochrome *c*, a significant decrease in the porphyrin emission peak at 610 nm was observed, indicative of protein binding. These emission intensity changes were plotted against cytochrome *c* concentration and fitted to a 1:1 binding model, yielding a dissociation constant (*K*_d_) of 32 nM, Figure 6. To verify that the neutral G4.0-OH **3** dendrimer alone does not bind the protein, a control titration was performed. This experiment utilized a solution of G4.0-OH **3** dendrimer with encapsulated Zn-THPP **5**, but without LC-Tyr **4**. As anticipated, no change in porphyrin emission intensity was observed with respect to increasing amounts of cytochrome *c*, confirming the absence of binding for the neutral unfunctionalized system, Figure 5. Despite the absence of negative charges in the G4.0-OH **3** self-assembled system (to bind to the positively charged surface of cytochrome *c*), the observed binding affinity was comparable to reported data for other surface-binding ligands. For instance, Jain and Hamilton reported *K*_d_ values between 20 and 120 nM for various porphyrins with up to eight terminal negative charges.^33^ Similarly, Wilson et al. observed dissociation constants ranging from 2 to 23 nM for a series of metal ligands, also possessing multiple terminal negative charges.^34^ Our previous results with the anionic G 3.5 CO_2_H dendrimer **2**, both with and without LC-Tyr **4**, yielded *K*_d_ values of 11 nM and 5.5 nM, respectively. Given that the neutral dendrimer scaffold **3** lacks negative charges, and LC-Tyr **4** does not have a terminal carboxylate (the amino acid is connected to the chain through its C-terminus), then the observed binding affinity must arise from cooperative interactions between other molecular features. These include π–π stacking, hydrogen bonding, and hydrophobic interactions contributed by the tyrosine linear chain.

**Figure 5:**
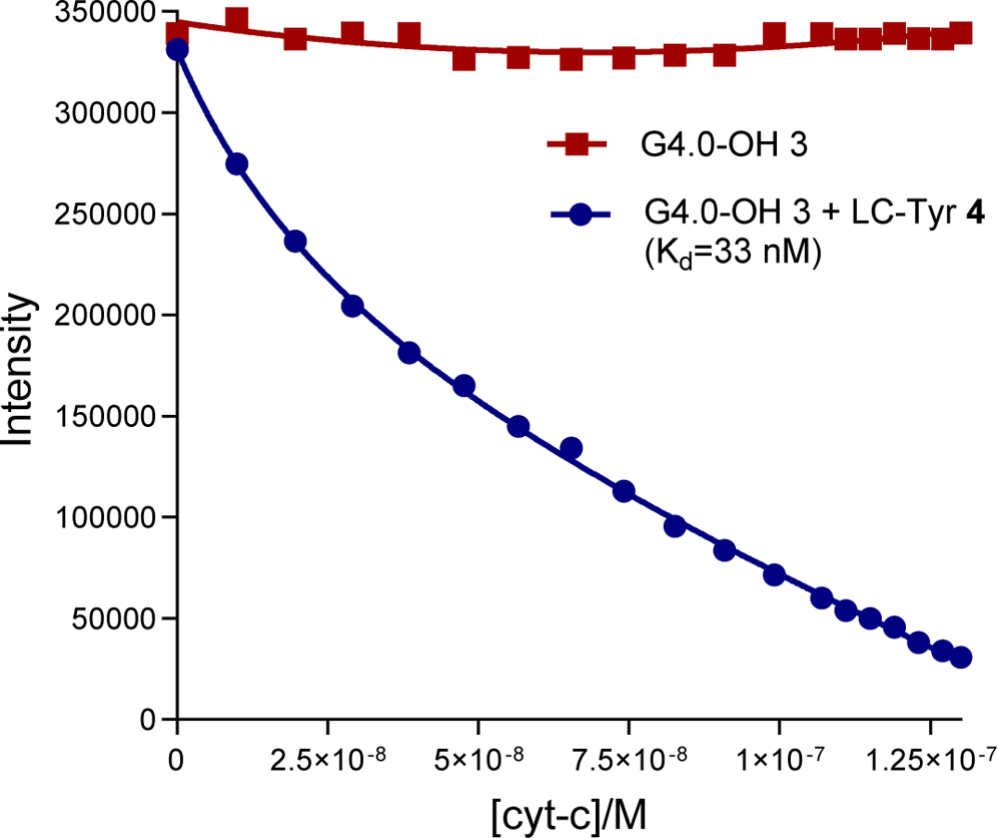
Titration plots of cytochrome *c* with the neutral nonfunctionalized complex using dendrimer **3** (square points). In this case binding is not detected. This is because the terminal OH groups are neutral and cannot interact electrostatically with the charged protein surface. When the tyrosine linear chain (LC-Tyr **4**) is added to give the neutral complex **1**, binding is observed (circle points). This is due solely to interactions between the protein surface and tyrosine’s aromatic, phenolic and amide functional groups. Plots show changes of intensity for the encapsulated Zn-THPP **5** peak at 615 nm (excitation at 410 nm) as cytochrome *c* is added.

### Control Experiments

To validate the proposed self-assembly mechanism for protein binding, a series of control experiments were conducted. These controls were designed to confirm that the individual components did not bind independently to cytochrome *c*, and that protein binding was contingent upon the formation of the dendrimer complex. These experiments mirrored the titration experiments described previously, but without the G3.5-CO_2_H dendrimer **2** (Figure 5). Due to the limited solubility of Zn-THPP **5**, the first control experiment, employing only Zn-THPP **5**, presented a challenge. However, using a saturated solution, a weak emission peak was observed. Upon the addition of cytochrome *c*, no quenching of this emission was observed, confirming that Zn-THPP **5** does not independently bind to the protein, Figure 6a. In a second control, LC-Tyr **4** was dissolved in the same saturated Zn-THPP **5** solution. Titration with cytochrome *c* again showed no evidence of emission quenching or binding, Figure 6b. Collectively, these results demonstrate that the presence of the dendrimer is essential for protein binding.

**Figure 6:**
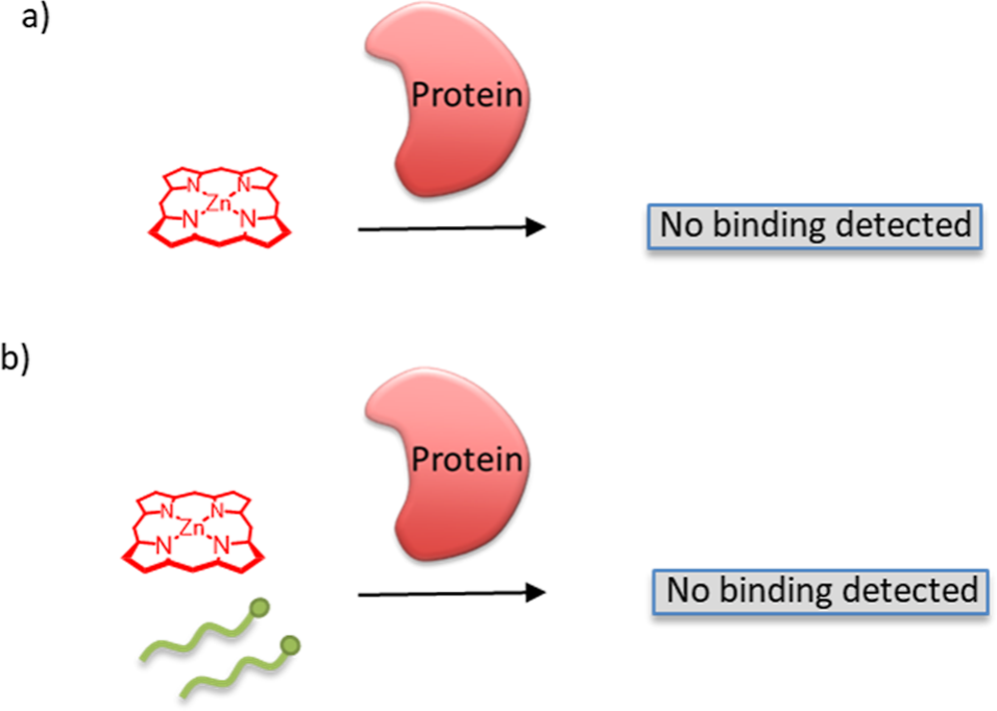
Schematic representation of the control experiments. (a) Control using just the porphyrin sensing unit Zn-THPP **5**, and (b) the control with targeting chain LC-Tyr **4** and porphyrin sensing unit Zn-THPP **5**.

## Conclusions

While covalent surface functionalization of macromolecules has shown promise for protein binding, achieving precise spatial control over ligand presentation remains a significant challenge. Specifically, controlling the three-dimensional arrangement of binding moieties, crucial for targeted protein interactions, is difficult. To overcome these limitations, we employed an orthogonal supramolecular self-assembly strategy. This approach utilizes a dendrimer scaffold as a platform to encapsulate linear amide chains terminated with tyrosine residues, known to enhance protein–protein binding affinity, along with a porphyrin sensing unit. Encapsulation was achieved through a synergistic combination of noncovalent interactions, including coordination, hydrophobic, electrostatic, and hydrogen bonding. Spectroscopic analysis using UV–visible spectroscopy confirmed successful encapsulation of both porphyrin and the linear amide chains. Encapsulation was evidenced by a red shift (bathochromic shift) in the porphyrin’s Soret band, indicating a change in its electronic environment. Additionally, a 5 nm bathochromic shift in the λ_max_ of the linear amide chains was observed. These observations confirm the successful incorporation of both species into the supramolecular assembly.

Initial protein binding assays, using cytochrome *c* as a model protein and a carboxylated dendrimer–porphyrin complex, demonstrated the dendrimer’s capacity for protein interaction, exhibiting a dissociation constant (*K*_d_) of 11 nM. Subsequent introduction of the tyrosine-terminated chains resulted in a significant increase in binding affinity, with a *K*_d_ of 5.5 nM. In both cases, the binding is strongly influenced by electrostatic interactions between the dendrimer’s carboxylate groups and the positively charged binding domain of cytochrome *c*. To isolate and quantify the binding contribution of the tyrosine-terminated chains, a neutral hydroxyl-terminated dendrimer scaffold was utilized. In the absence of the linear amide chains, the hydroxyl-terminated dendrimer–porphyrin complex did not bind to cytochrome *c*. Conversely, the addition of the tyrosine-terminated chains yielded a *K*_d_ of 32 nM. The absence of terminal carboxylate groups in the linear chains, due to tyrosine’s incorporation through its carboxylic acid group, eliminates electrostatic interactions as the primary binding mechanism. Thus, the observed binding affinity is attributed to the aromatic and phenolic functionalities of tyrosine.

The methodology described employs a modular dendrimer-based platform for protein detection. By selectively exchanging binding and/or sensor moieties on a common dendrimer scaffold, or conversely, utilizing consistent binding/sensor groups across a range of dendrimer architectures, diverse protein targets can be addressed. This modularity facilitates the generation of a wide array of dendrimer constructs for targeting various proteins. More importantly, this approach offers a solution to problems related to a lack of selectivity with respect to problematic off target effects in biological and medicinal applications. Future experiments will focus on developing methods to fix or trap the linear chains within the dendrimer scaffold, which could include cross-linking or photoactivation. In addition to combining multiple targeting groups, future work will also investigate dendrimer scaffolds of differing sizes and binding studies using a variety of additional proteins.

## Experimental Section

### Materials

All reagents and solvents were obtained from commercial sources (primarily Sigma-Aldrich) and were used without further purification. Dry solvents were obtained from the University of Sheffield Chemistry Department Grubbs solvent dispensing system. Dendrimers were synthesized and characterized using previously reported methods.^1^ All glassware was cleaned and dried in an oven overnight (100 °C) before use.

### UV Spectrophotometry

Absorbance was recorded on an Analytic Jena AG Specord s600 UV/vis spectrometer and analyzed using WinASPECT.

### Infrared Spectroscopy

IR spectra were recorded using a PerkinElmer UATR Infrared spectrometer. Spectra were analyzed with Spectrum100 software.

### Fluorescence Spectroscopy

Emission was recorded on a PerkinElmer Fluoromax-4 Spectrofluorometer at 25 °C and spectra analyzed with FluorEssence V3software.

### NMR Spectroscopy

All NMR samples were prepared using deuterated solvents supplied by Sigma-Aldrich. ^1^H NMR and ^13^C NMR spectra were recorded using a Bruker AV1400 MHz machine. Chemical shifts are quoted using ppm and referenced to residual solvent signals, coupling constants are quoted in Hertz. The NMR spectra were analyzed using Topspin 3.0 NMR software.

### Mass Spectrometry

For dendrimers, a Bruker reflex III MALDI-ToF mass spectrometer was used. For all other samples, a Waters LCT Premier XE spectrometer, and electrospray ionization (ES) was used.

### Accurate Mass Spectrometry

A high resolution Agilent 6530 Accurate-Mass Q-TQF spectrometer and electrospray ionization was used.

## Synthesis

### Cbz-Protected Amide Chain (**8**)

*n*-Propylamine **6** (1.30 g, 0.022 mol), *N*-Cbz-β-alanine **7** (5.00 g, 0.022 mol) and DMAP (5.38 g, 0.044 mol) were dissolved in DCM (150 mL). EDC·HCl (4.22 g, 0.022 mol) and triethylamine (6.68 g, 0.066 mol) were added to the mixture. The mixture was allowed to react under nitrogen condition for 24 H. The crude product was washed with brine (100 mL × 3), and 2 M of HCl, and the aqueous layers were backwashed with DCM. The organic layers were collected and dried with Mg_2_SO_4_. The solvent was concentrated at reduced pressure and dried under a high vacuum to give Cbz chain **8** as a white powder in 81% yield: FTIR (ν_max_/cm^–1^), 3324, 3293.2 (N–H stretch), 3078 (C–H aromatic), 2959, 1684, 1644, 1528, 1227, 1028; ^1^H NMR (400 MHz; CDCl_3_): 7.30 (5H, *m*,CHAr), 6.23 (1H, *t*, CONHCH_2_), 5.75 (1H, *t*, CONHCH_2_), 5.06 (2H, *s*, CH_2_Ar), 3.45 (2H, *m*, NHCH2 CH2), 3.17 (2H, *m*, NHCH_2_ CH_2_), 2.40 (2H, *t*, NHCH_2_ CH_2_), 1.48 (2H, *m*, CH_2_CH_3_), 0.87 (3H, *t*, CH_2_ CH_3_); ^13^C NMR (100 MHz; CDCl_3_): 171.2, 156.8, 136.59, 128.12, 66.54, 41.16, 37.29, 36.16, 23.49, 11.83; Mass spec (ES) 264 (M^+^), C_14_H_20_N_2_O_3_, 264 (calcd).

### Deprotected Amide Chain (**9**)

Cbz-protected chain **8** (4.00 g, 0.015 mol), was dissolved in 25 mL of methanol. A catalytic amount of Pd/C (5% on carbon, 0.40 g) was added to a round-bottomed flask followed by the Cbz solution under nitrogen. Then, the reaction mixture was kept under stirring and an H_2_ balloon was placed on top of the flask. The mixture was filtered over Celite, and the filtrate was concentrated under reduced pressure to give **9** as a viscous yellow oil in 84% yield. FTIR (ν_max_/cm^–1^), 2978, 1645, 1558; ^1^H NMR (400 MHz; CDCl_3_): 3.19 (2H, *t*, CH_2_ CH_2_NH), 3.01 (2H, *t*, CH_2_ CH_2_NH_2_), 2.34 (2H, *t*, CH_2_CH_2_NH_2_), 1.50 (2H, *m*, CH_3_ CH_2_), 0.91 (3H, *t*, CH_2_ CH_3_); ^13^C NMR (100 MHz; CDCl_3_): 172.2, 50.3, 41.1, 37.9, 22.73, 11.4; Mass spec (ES) 131 (MH^+^), C_6_H_15_N_2_O, 131 (calcd).

### Cbz-Protected Diamide Chain (**10**)

The title compound was synthesized using the method described above for the Cbz-protected amide chain **8**, using the following: (4.26 g, 0.032 mol) of the deprotected chain **9**, *N*-Cbz-β-alanine (6.69 g, 0.032 mol) and DMAP (7.33 g, 0.06 mol) were dissolved in THF (150 mL). EDC·HCl (5.73 g, 0.032 mol) and triethylamine (9.19 g, 0.09 mol) were added to the mixture. The mixture was stirred under nitrogen for 24 h. After work up, the Cbz-protected diamide chain **10** was obtained as a white solid in 66% yield. FTIR (ν_max_/cm^–1^), 3324, 3293, 3005, 2911, 1680, 1629, 1528, 1227; ^1^H NMR (400 MHz; CDCl_3_): 7.36 (5H, *m*, CHAr), 5.11 (2H, *s*, CH_2_Ar), 3.49 (4H, *m*, NHCH_2_ CH_2_), 3.21 (2H, *m*, NHCH_2_ CH_2_), 2.40 (4H, *t*, NHCH_2_ CH_2_), 1.54 (2H, *m*, CH_2_CH_3_), 0.93 (3H, *t*, CH_2_ CH_3_); ^13^C NMR (100 MHz; CDCl_3_): 171.5, 171,4, 156.4, 136.5, 128.1, 66.5, 41.3, 37.1, 35.4, 22.8, 11.4; Mass spec (ES) 336 (MH^+^), C_17_H_26_N_3_O_6_, 336 (calcd).

### Deprotected Diamide Chain (**11**)

The title compound was synthesized using the method described above for the deprotected chain **9**,using the following amounts: Cbz-protected chain **10** (5.50 g, 0.016 mol) and Pd/C (5% on carbon, 0.50 g). After work up, the amide chain **11** was isolated as a viscous yellow oil in 96% yield. FTIR (ν_max_/cm^–1^), 2938, 1645, 1555, 1178, 1130; ^1^H NMR (400 MHz; CDCl_3_): 3.56 (2H, *q*, CH_2_ CH_2_NH), 3.22 (2H, *q*, CH_2_ CH_2_NH), 3.01 (2H, *t*, CH_2_ CH_2_NH), 3.01 (2H, *t*, CH_2_ CH_2_NH_2_), 2.43 (2H, *t*, CH_2_CH_2_NH), 2.32 (2H, *t*, CH_2_CH_2_NH_2_), 1.51 (2H, *m*, CH_2_CH_3_), 0.94 (3H, *t*, CH_2_ CH_3_); ^13^C NMR (100 MHz; CDCl_3_): 172.7, 171.43, 5.8, 41.2, 38.9, 38.2, 36.0, 35.4, 22.8, 11.4; Mass spec (ES) 202 (MH^+^), C_9_H_20_N_3_O_2_, 202 (calcd).

### Cbz-Protected Tyrosine Linear Chain **12**

Amide chain **11** (3.26 g, 0.016 mol), Cbz-l-tyrosine (5.04 g, 0.016 mol) and DMAP (3.90 g, 0.032 mol) were dissolved in THF (150 mL). EDC·HCl (3.06 g, 0.016 mol) and triethylamine (4.85 g, 0.048 mol) were added to the mixture. The mixture was stirred under nitrogen for 24 h. The solution was filtered and the solvent removed by rotary evaporation. The crude Cbz protected product was washed with brine (100 mL × 3), and 2 M of HCl, and the aqueous layers were backwashed with DCM. The organic layers were collected and dried with Mg_2_SO_4_. The solvent was concentrated at reduced pressure and dried under a high vacuum to give the protected tyrosine chain as a light-yellow powder in 66% yield. FTIR (ν_max_/cm^–1^), 3288 (N–H, stretch), 3100 (C–H aromatic), 2956 and 2877 (C–H), 1689 and 1638 (C=O stretch), 1542, 1513 (N–H bend), 1365 (O–H phenol), 1258 (C–N); ^1^H NMR (400 MHz; CD_3_OD): 7.31 (5Η, *m*, CHAr), 7.05 (2H, *d*, *m*-CHAr), 6.71 (2H, *d*, *o*-CHAr), 5.04 (2Η, *d*, CH_2_Ar), 4.25 (1H, *m*, CHNH), 3.41 (2H, *t*, NHCH_2_), 3.29 (2H, *t*, NHCH_2_), 3.13 (2H, *t*, NHCH_2_), 2.98 (1H, dd, diastereotopic CH_2_), 2.80 (1H, diastereotopic CH_2_), 2.38 (4H, *tt*, NHCH_2_ CH_2_), 1.50 (2H, *m*, CH_2_CH_3_), 0.91 (3H, *t*, CH_2_ CH_3_); ^13^C NMR (100 MHz; MeOD): 172.9, 129.9, 128.1, 127.6, 127.2, 114.8, 106.8, 66.2, 56.9, 40.85, 37.0, 36.4, 35.6, 35.3, 35.2, 22.2, 10.3; Mass spec (ES) 499 (MH^+^), C_26_H_35_N_4_O_6_, 499 (calcd).

### Tyrosine Linear Chain—LC-Tyr **4**

The Cbz protected chain was deprotected using the same deprotection method described for **9** using the following amounts; Cbz-protected tyrosine chain (3.60 g, 7.20 mmol) and Pd/C (5% on carbon, 0.30 g). After workup, the final tyrosine functionalized linear chain **4** was obtained as a light-yellow powder in a 73% yield. FTIR (ν_max_/cm^–1^), 3288 and 3086 (N–H), 2936 (C–H), 1634 (C=O), 1536 and 1512 (N–H), 1185 and 1138 (C–N); UV absorbance (MeOH) λ_max_ (nm) 275, 227; ^1^H NMR (400 MHz; CD_3_OD): 7.03 (2H, d, *J* = 8 Hz, *m*-CHAr), 6.73 (2H, *d*, *J* = 8 Hz, *o*-CHAr), 3.84 (1H, *t*, *J* = 7 Hz CH_2_ CHNH_2_), 3.40 (4H, *m*, CH_2_ CH_2_NH), 3.14 (2H, *t*, *J* = 7 Hz, CH_2_ CH_2_NH), 2.90 (1H, dd, *J* = 7 and 12 Hz, diastereotopic CH_2_), 2.74 (1H, dd, J = 7 and 12 Hz, diastereotopic CH_2_), 2.83 (2H, *t*, *J* = 7 Hz CH_2_CH_2_), 2.31 (2H, sx, *J* = 7 Hz, NHCH_2_ CH_2_), 1.52 (2H, *m*, CH_3_ CH_2_), 0.93 (3H, *t*, *J* = 7 Hz, CH_2_ CH_3_); ^13^C NMR (100 MHz; MeOD): 175.2, 172.3, 156.0, 130.0, 127.9, 114.9, 56.2, 40.8, 40.1, 35.8, 35.4, 35.2, 35.2, 22.2, 10.3, Mass spec (ES) 365 (MH^+^). HRMS-ES, calcd for C_9_H_19_N_3_O_2_ [MH+]: 365.2183; found, 365.2189.

### Zinc Tetrahydroxyphenylporphyrin—Zn-THPP (**5**)^1,2^

Freshly distilled pyrrole (12.51 g, 180 mmol) and 4-hydroxylbenzaldehyde (30.0 g, 120 mmol) were refluxed in propionic acid (500 mL) for 24 h. The mixture was allowed to cool to room temperature and left for 2 h at −5 °C. The crude product precipitated and was collected by filtration. The solid was washed with cold propionic acid and then recrystallized from ethanol to give the free-base porphyrin as a purple solid. The free-base porphyrin (2.0 g, 2.0 mmol) and an excess of zinc acetate-dihydrate (2.0 g) were refluxed in 100 mL of DCM for 10 min. The solution was filtered and evaporated and the crude product recrystallized from DCM/hexane to give Zn-THPP **5** as purple crystals in a yield of 2%. UV (MeOH) λ_max_ (nm) 425, 595, 660; FTIR (ν_max_/cm^–1^), 2923, 3245, 2924,1609, 1465; ^1^H NMR (400 MHz; DMSO): 9.97 (s, 4H), 8.84 (s, 8H), 7.96 (d, J 8.50), 7.18 (d, 8H, J 8.50); ^13^C NMR (100 MHz; CDCl_3_): 134.5, 127.6, 126.5, 120.4, 11.8; MS (ES), 743 (MH^+^), C_44_H_28_N_4_O_4_Zn, 743 (calcd).

## Supplementary Material



## Notes

Binding titration data and spectra, encapsulation experiments, along with the ^1^H and^13^C NMR spectra of the linear chain and intermediates (PDF).
