# Supplementary material for: An Orthogonal Supramolecular Approach toward Protein Binding and Protein Sensing Using Dendrimers as Scaffolds for the Noncovalent Assembly of Binding and Sensing Groups
Source: ACS Mater Au. 2025 Aug 8;5(5):849–57. doi: 10.1021/acsmaterialsau.5c00049 (PMC12426775; doi:10.1021/acsmaterialsau.5c00049)
Supplement: Supplementary file 1 [file mg5c00049_si_001.pdf]

SUPPORTING INFORMATION

An orthogonal supramolecular approach towards protein binding and protein sensing using dendrimers as scaffolds for the non-covalent assembly of binding and sensing groups.

Azrah Aziz, Lance J. Twyman, Amal Al Ageel, Ibrahim O. Althobaiti and Abdullah N. Alotaibi*

School of Mathematical and Physical Sciences, Chemistry, University of Sheffield, Dainton Building, Brook Hill, Sheffield, South Yorkshire, UK. S3 7HF.

* Corresponding Author; Email; l.j.twyman@sheffield.ac.uk

Protein binding titration

A stock solution of the macromolecular ligand was prepared by adding dendrimer, linear chain and Zn-THPP to methanol. The methanol was removed and phosphate buffer (pH 7.4, 0.01M) was added to the resulting paste to give a solution that was 1.0 μM in dendrimer and Zn-THPP and 10 μM in linear chain. For control experiments the same procedure was used, but one or more components were removed. To ensure the titration was performed under constant concentration of Zn-THPP, the stock solution was also used as the solvent to make a 1.0 μM Cytochrome-c solution. 1.5 mL of the stock solution was added to a quartz cuvette and placed into a fluorimeter. Aliquots (20 μL) of the cytochrome-c solution were then added and the intensity of the emission peak at 610 nm (excitation at 410 nm) monitored after each addition. The data was plotted with respect to the concentration of cytochrome-c and curve fitting analysis used to fit the experimental data to a 1:1 binding model (Graphpad prism 7.0).

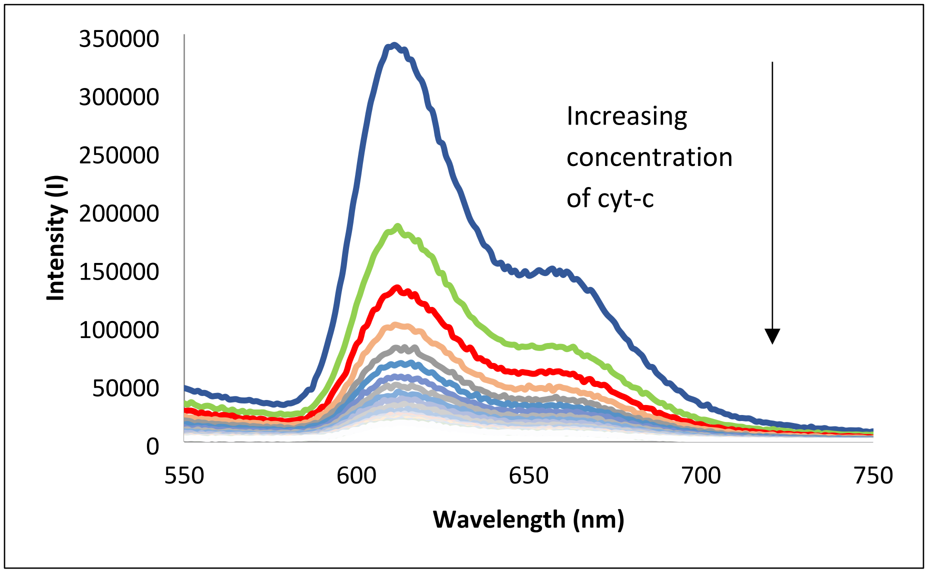

Figure S1: *Emission spectra for the titration of 20 μL of Cyt-c to a solution that was 1.0 μM in carboxylate G3.5-COOH* ***2*** *and ZnTHPP complex (control with NO linear chain).*

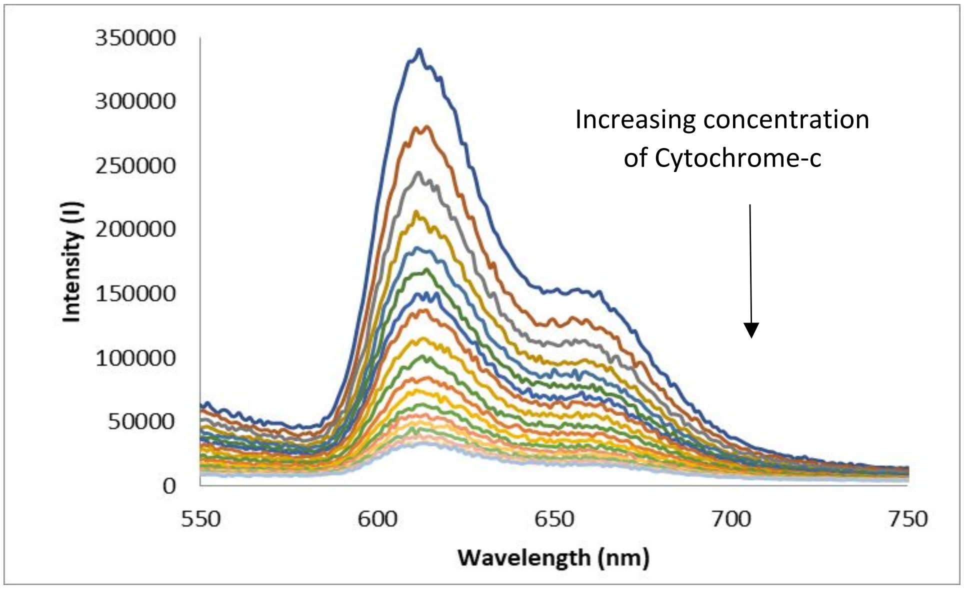

Figure S2: *Emission spectra for the titration of 20 µL of Cyt-c to a solution that was 1.0 µM in neutral G3.5-OH and ZnTHPP **5** complex **1** and 10.0 µM in LC-Tyr **4**.*

Encapsulation of LC-Tyr *4* chain and Zn-THPP within PAMAM dendrimers.

Dendrimer, Zn-THPP and LC-Tyr **4** were dissolved in methanol (100 mL) to give concentrations 1.0×10^{-5} M, 1.0×10-5 M and 1.1×10^{-4} M respectively (a 1:1:10 ratio). The methanol was then completely removed using a rotary evaporator and then a high vacuum to give a coprecipitate. 1.0 L of tris buffer (pH 7.46, 0.01M) was then added to the coprecipitate, giving a solution that was 1.0×10^{-6} M in dendrimer and Zn-THPP and 1.1×10^{-5} M in LC-Tyr **4**. The binding and loading of Zn-TPP was confirmed using UV, which showed a clear peak that was 13 nm shifted with respect to the unbound porphyrin (412 nm to 425 nm shift, due to complexation of the Zinc with the internal amines of the dendrimer). The amount of *encapsulated* LC-Tyr **4** was calculated by subtracting the concentration of a saturated solution of LC-Tyr **4** (*free* LC-Tyr chain **4** = 0.51×10^{-5} M). The loading of LC-Tyr **4** within the dendrimer was determined by dividing the

concentration of the dendrimer by the concentration of *encapsulated* LC-Tyr **4**. The UV spectrum of encapsulated (within G 3.5 COOH dendrimer **2**) and free LC-Tyr **4** is shown below.

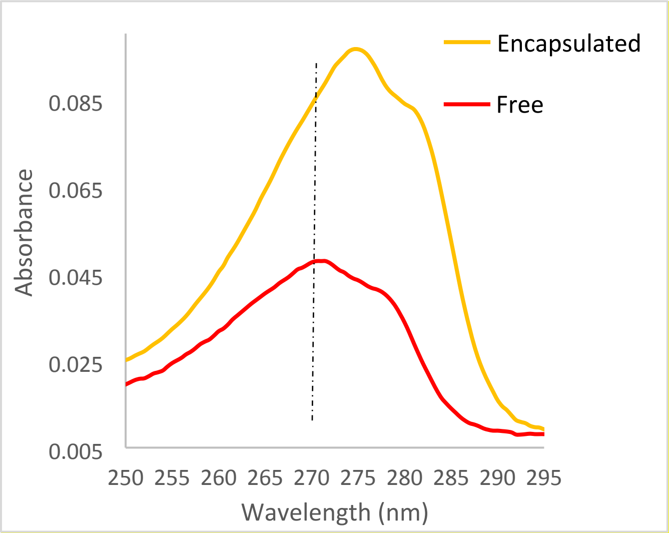

Figure S3: *UV spectra (buffer-pH 7.4) of the free and encapsulated LC-Tyr **4** chain.*

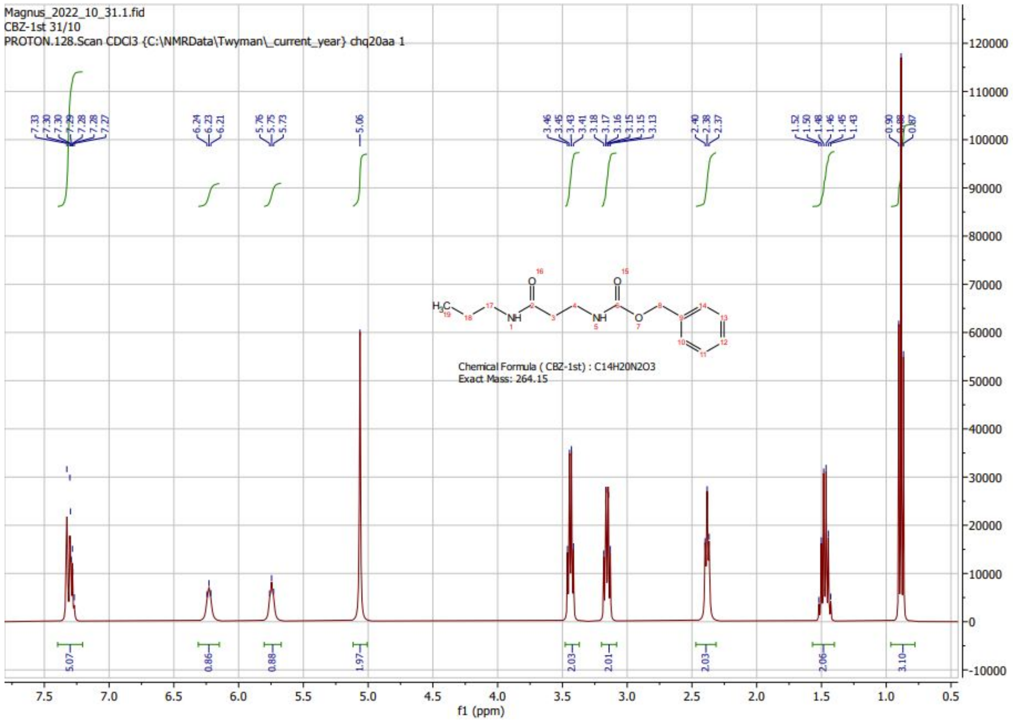

Figure S4: *^{1}H NMR spectra of compound **8** recorded at 400MHz and referenced to residual solvent ($CDCl_3$).*

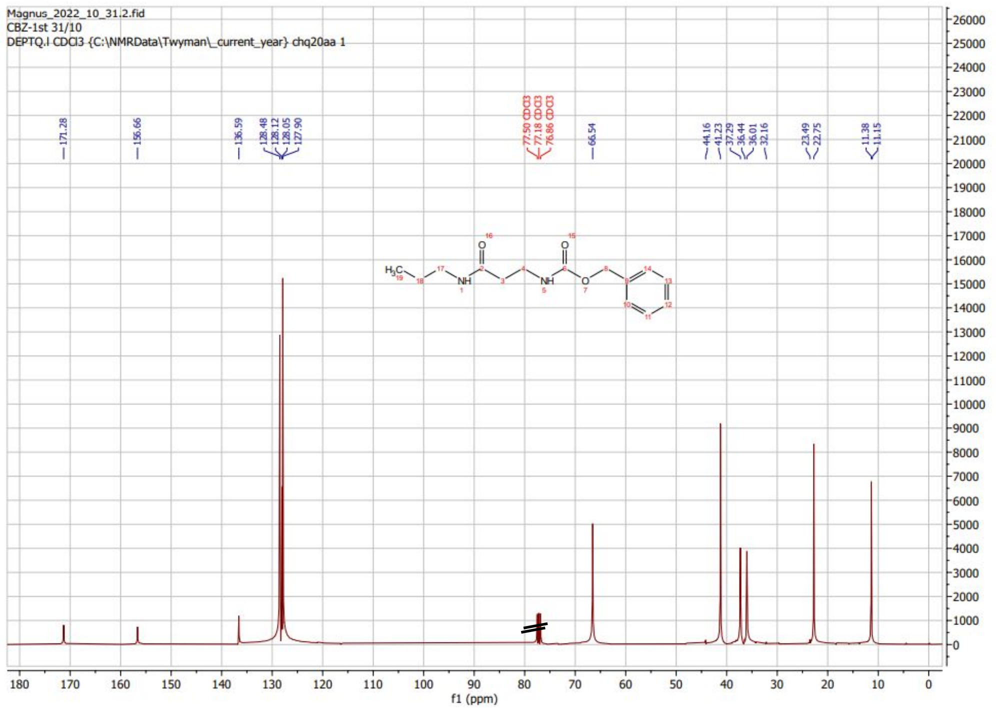

Figure S5: *^{13}C NMR spectra of compound* ***8*** *recorded at 100MHz and referenced to residual solvent ($CDCl_3$).*

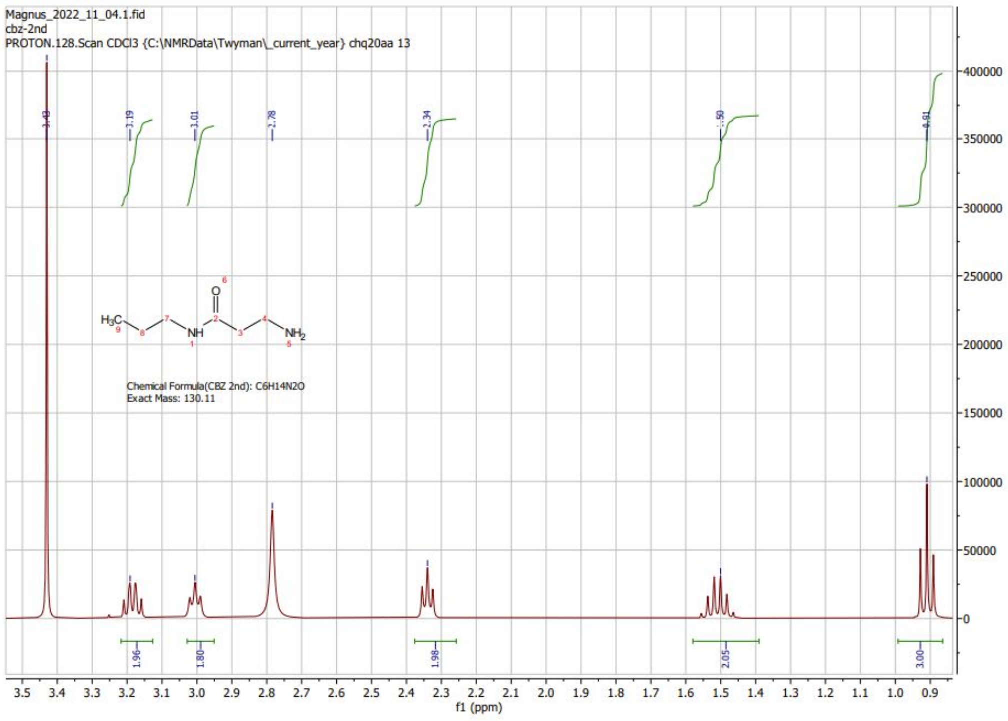

Figure S6: *^{1}H NMR spectra of compound* ***9*** *recorded at 400MHz and referenced to residual solvent ($CDCl_3$).*

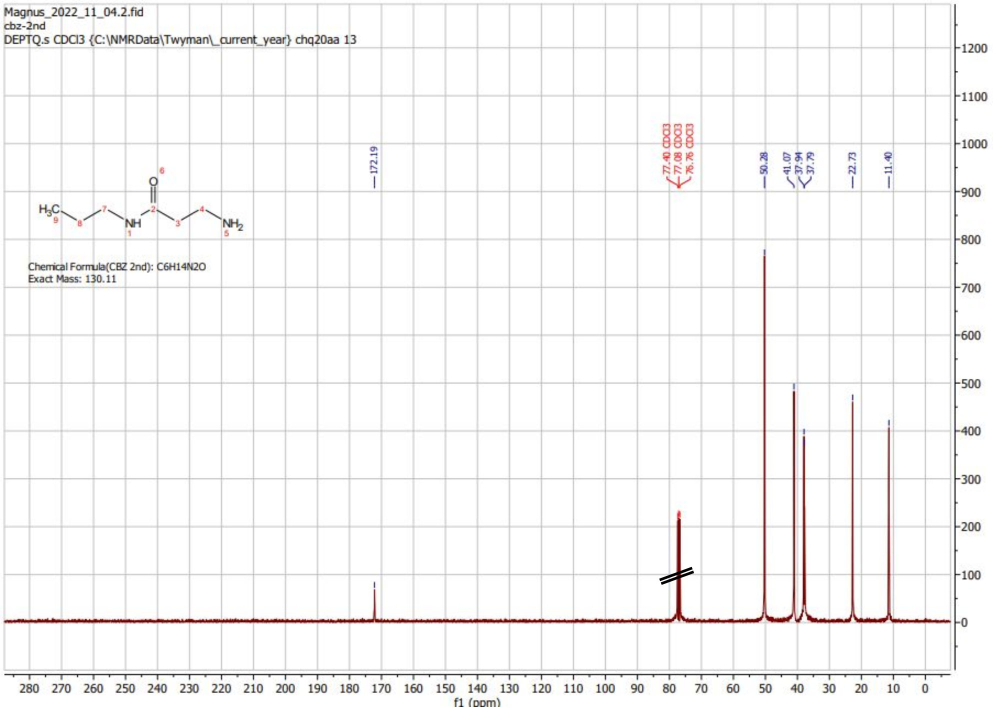

Figure S7: *^{13}C NMR spectra of compound **9** recorded at 100MHz and referenced to residual solvent ($CDCl_3$).*

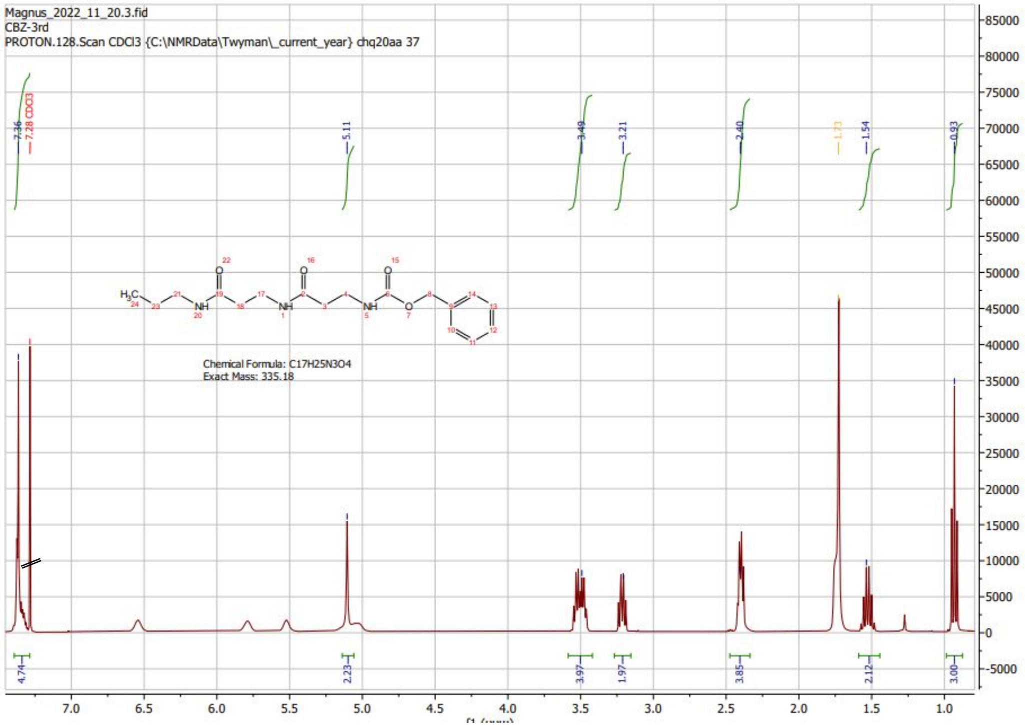

Figure S8: *^{1}H NMR spectra of compound **10** recorded at 400MHz and referenced to residual solvent ($CDCl_3$).*

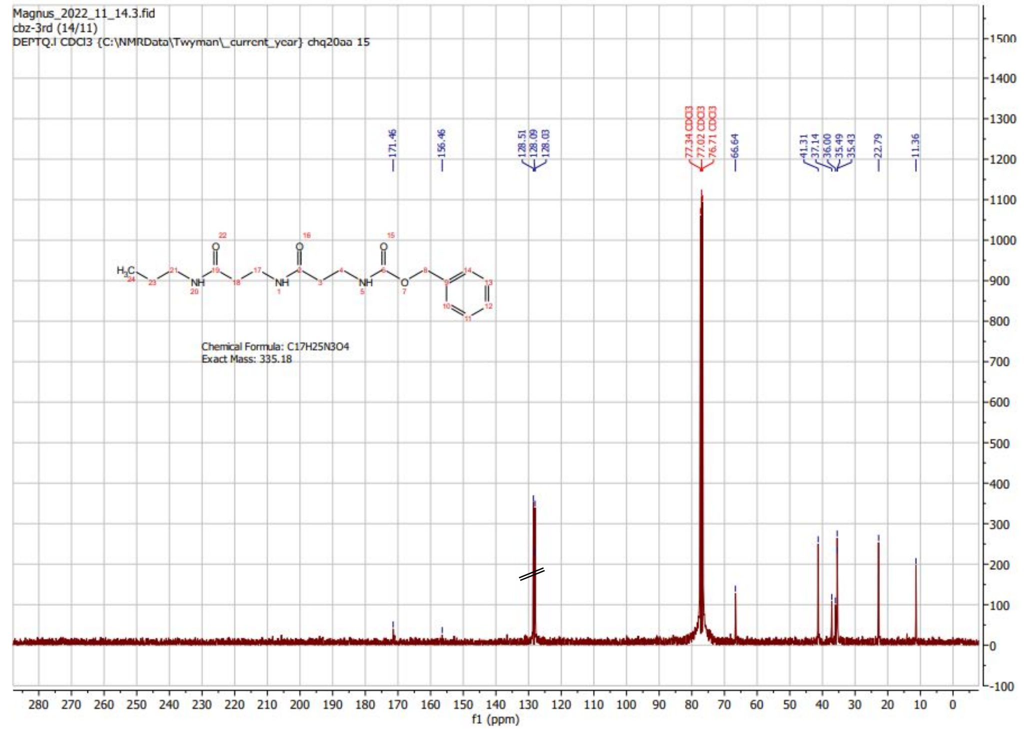

Figure S9: *^{13}C NMR spectra of compound **10** recorded at 100MHz and referenced to residual solvent ($CDCl_3$).*

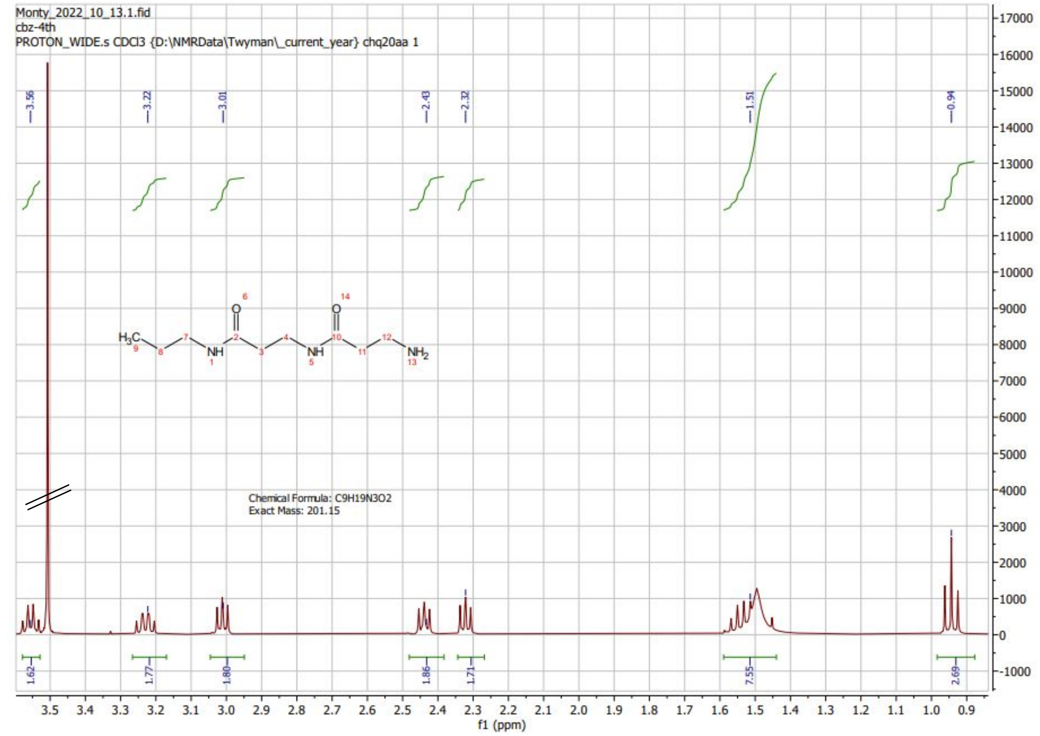

Figure S10: *^{1}H NMR spectra of compound **11** recorded at 400MHz and referenced to residual solvent ($CDCl_3$).*

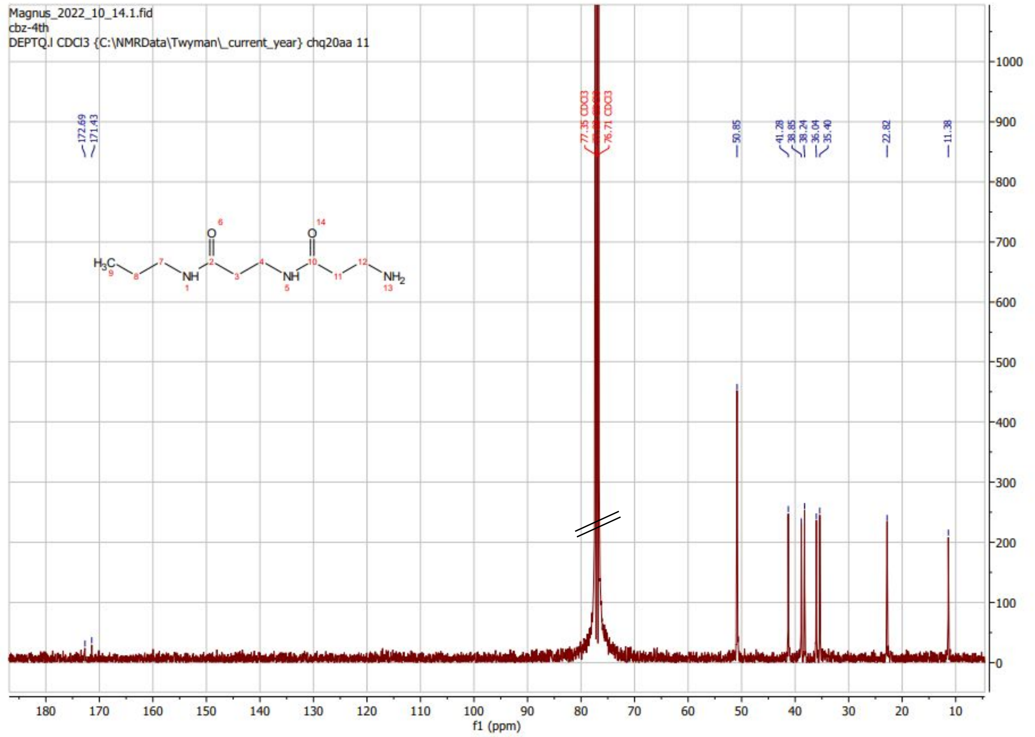

Figure S11: ^{13}C *NMR spectra of compound* ***11*** *recorded at 100MHz and referenced to residual solvent (CDCl$_3$).*

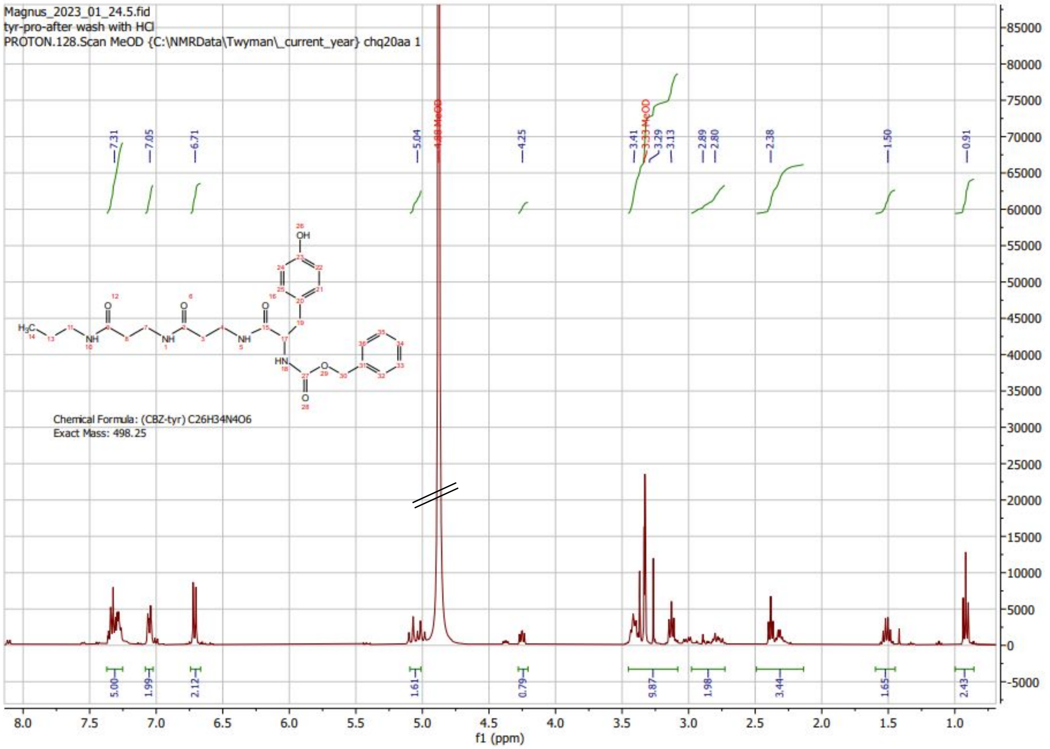

Figure S12: ^{1}H *NMR spectra of compound* ***12*** *recorded at 400MHz and referenced to residual solvent (CD$_3$OD).*

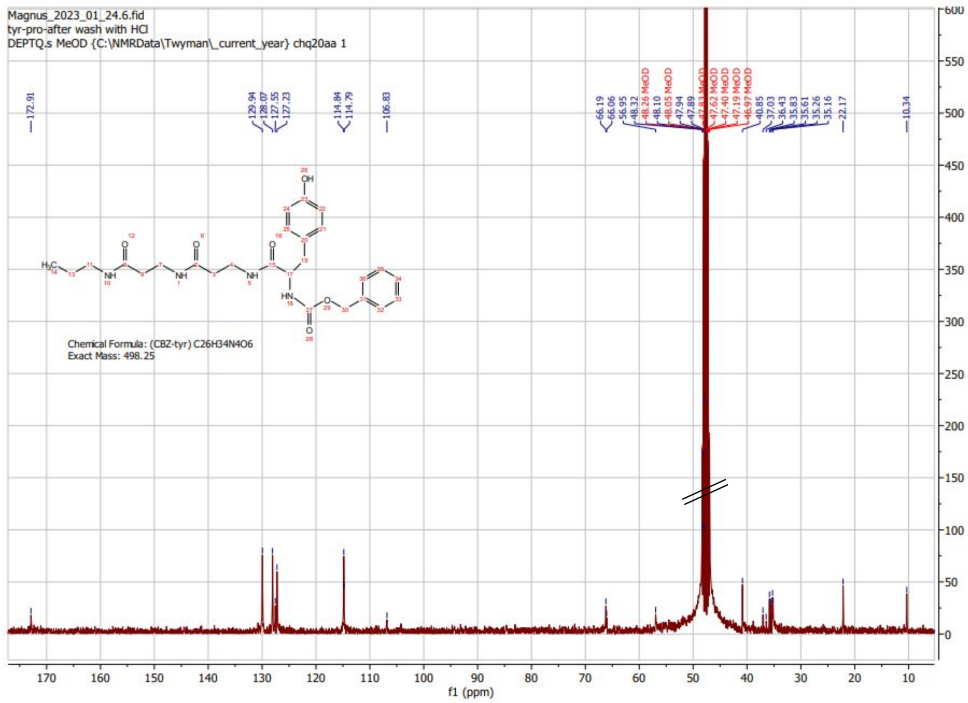

Figure S13: ^{13}C *NMR spectra of compound* ***12*** *recorded at 100MHz and referenced to residual solvent* (CD_3OD).

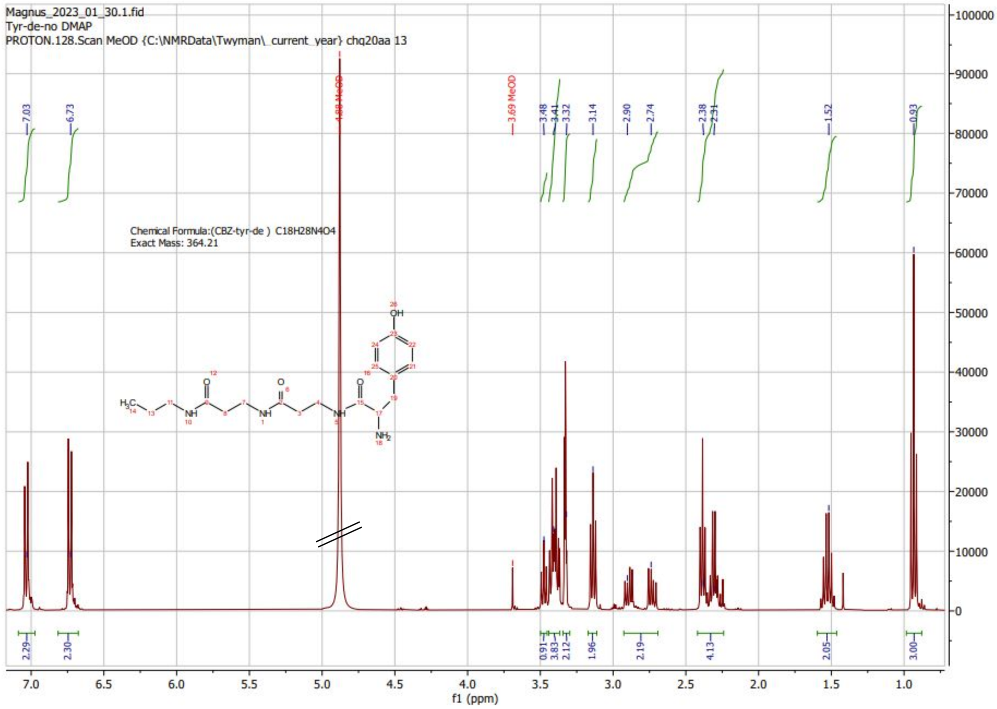

Figure S14: ^{1}H *NMR spectra of LC-Tyr* ***4*** *recorded at 400MHz and referenced to residual solvent* (CD_3OD).

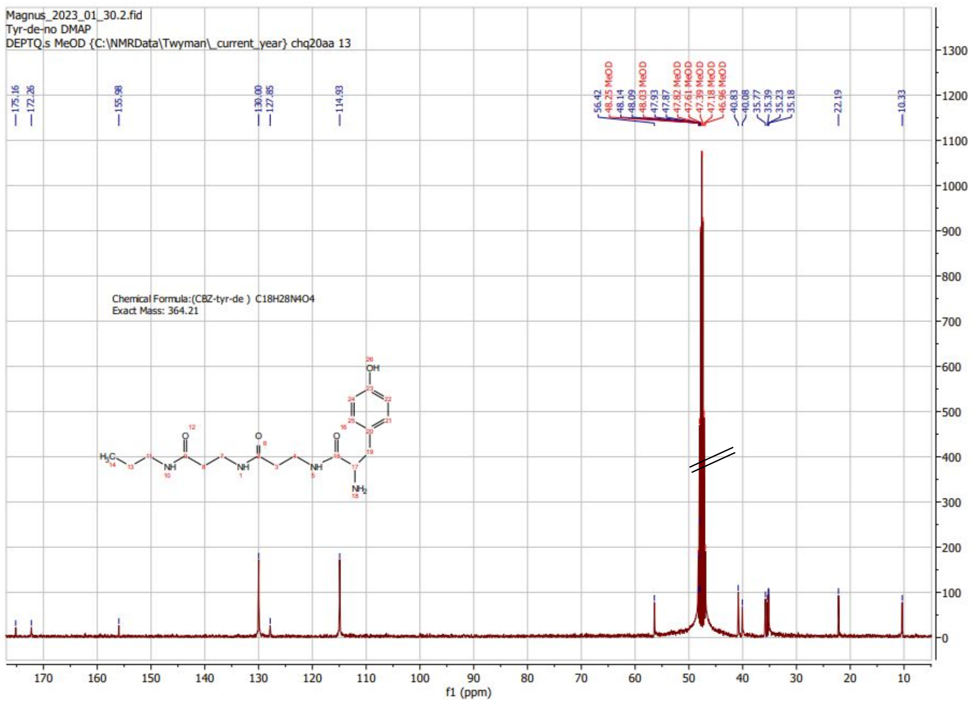

Figure S15: *^{13}C NMR spectra of LC-Tyr **4** recorded at 100MHz and referenced to residual solvent (CD_3OD).*

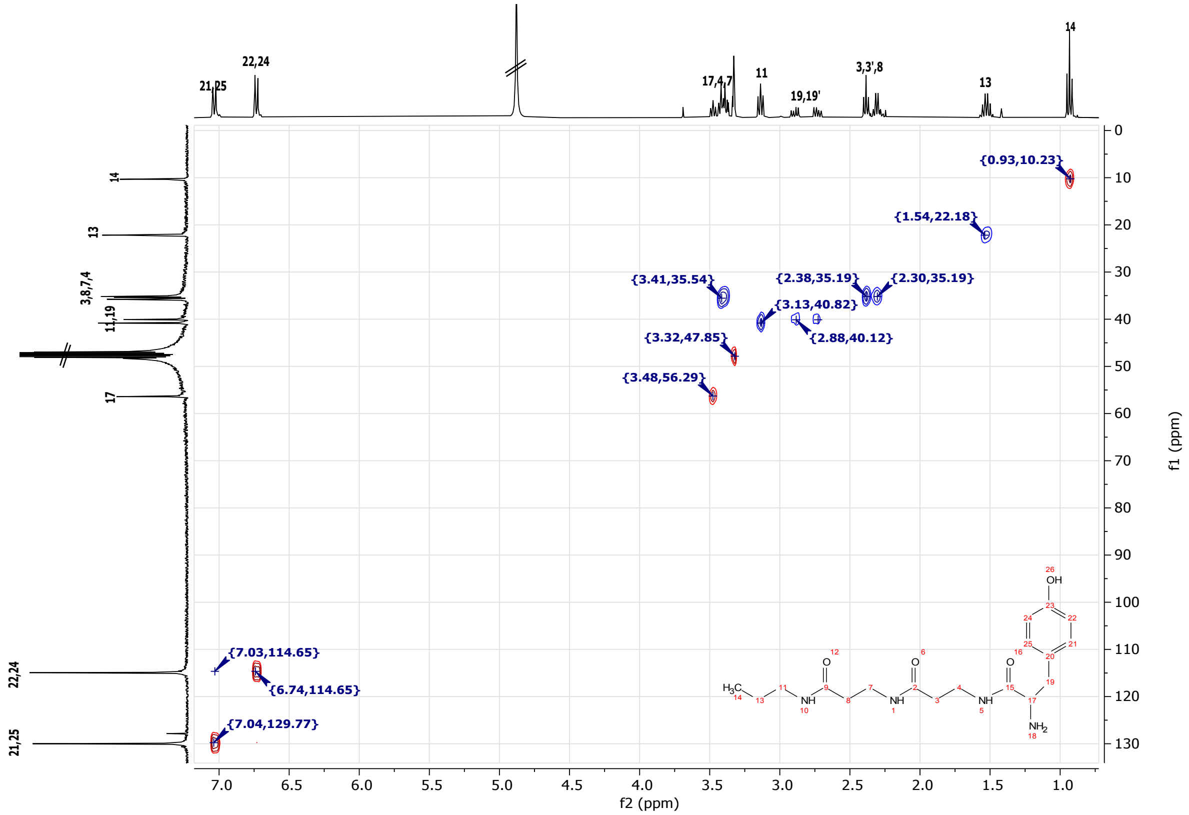

Figure S16: *2D ^{1}H/ ^{13}C HSQC NMR of LC-Tyr **4** (in CD3OD)*

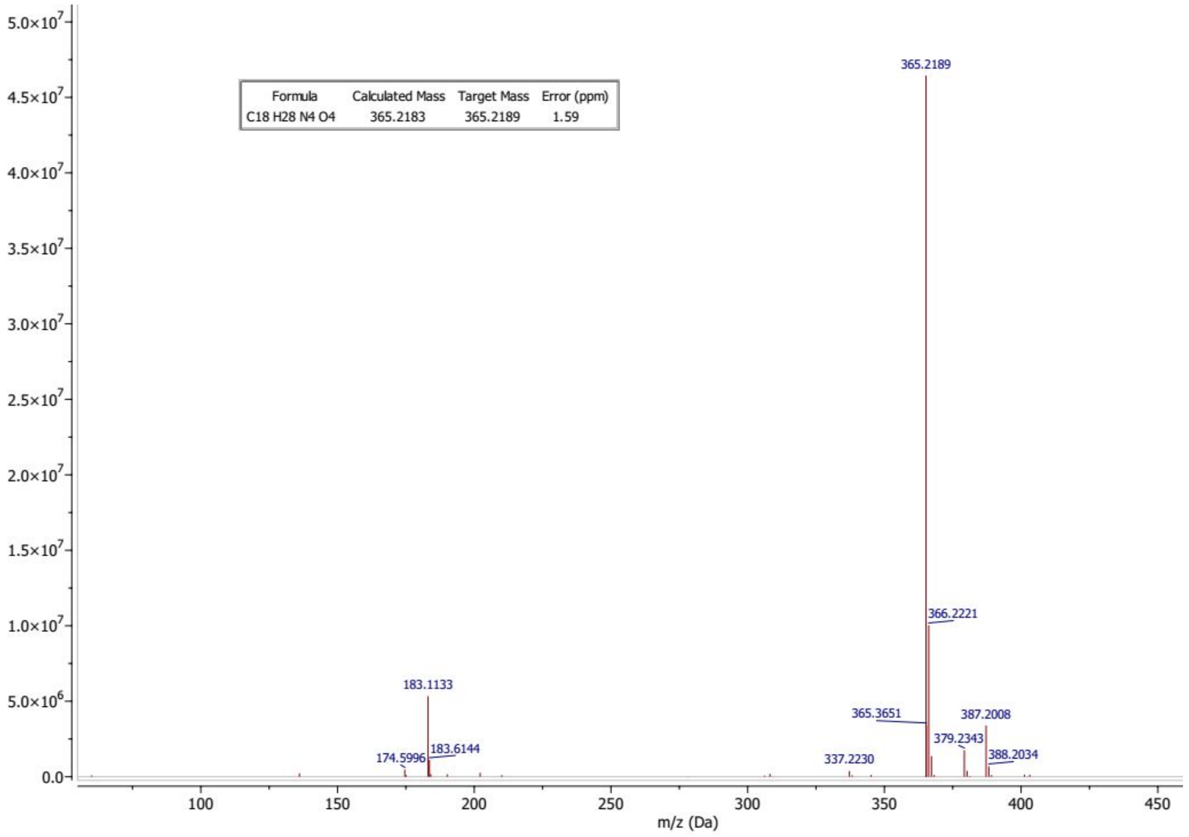

Figure S17*: HIGH RES MS of LC-Tyr **4** chain.*

References

1. A. Kadhim, L. K. McKenzie, H. E. Bryant and L. J. Twyman, *Molecular Pharmaceutics*, 2019, *16(3)*, 1132-1139.
2. S. Shanmugathasan, C. Edwards and R. W. Boyle, *Tetrahedron*, 2000, **56**, 1025–1046
